# Supplementary material for: Associations between impulsivity and self-care adherence in individuals diagnosed with Type 2 or prediabetes
Source: PLoS One. 2022 Mar 10;17(3):e0263961. doi: 10.1371/journal.pone.0263961 (PMC8912230; doi:10.1371/journal.pone.0263961)
Supplement: Attachment — Submitted filename: PONE-D-21-06140 Response to Reviewers.docx [file pone.0263961.s002.docx]

PONE-D-21-06140

Associations between impulsiveness and self-care adherence in individuals diagnosed with type 2 or prediabetes

PLOS ONE

We would like to thank the Reviewers and Editor for their careful review of our manuscript. Per their comments, offer our response (in ***italicized bold*** font) to their suggestions in our revised manuscript:

Reviewer #1:

Abstract

“have never been measured with participants”. This statement is vague and incorrect. Please see Hadj-Abo et al. (2020) who have recently examined these relationships in participants with diabetes.

***This statement is correct – Hadj-Abo et al. (2020) only measured impulsivity using the BIS-11, whereas we used both the BIS-11 and the delay discounting task (i.e., different impulsivity constructs). Additionally, Hadj-Abo et al. (2020) only recruited participants diagnosed with Type 2 diabetes, whereas we recruited individuals diagnosed with either Type 2 or prediabetes.***

delete “personality”

***Done***

Line 27 and throughout – Use the term ‘impulsivity’ instead of ‘impulsiveness’ unless referring specifically to the BIS.

***Done***

Introduction

As PLOS One is an interdisciplinary journal, consider expanding your definition of diabetes to include a brief discussion on the distinction between Type 1, Type 2, and prediabetes. Based on the information currently available in the introduction, it is not clear why this study has chosen to focus only on Type 2 and prediabetes.

***We have added additional information about the difference between the different diabetes subtypes and why we are choosing to only focus on Type 2 and prediabetes for this study.***

Remove apostrophe from pancreas.

***Done***

Consider including the reference at the end of the sentence to also support the statement of “multiple health complications”. Explicitly listing some of these complications may also help justify the importance of this topic.

***We have added 7 additional health complications arising from diabetes and provided a reference.***

Consider a different term for “behavioral treatment”. For example, “behavioral interventions” or “behavioral management strategies” may better capture the actions required to effectively manage diabetes and prevent health complications.

***We changed it to “behavioral interventions”.***

Include reference for “in addition to pharmacological treatments”.

***We have added this reference.***

Please expand your definition for “self-care plan complexity”. It is unclear exactly what this term refers to.

***We have added additional examples of self-care plan complexity, which are also measured in the current study via the SDSCA.***

Include ‘and’ between “organize execute”

***Done***

Given that self-efficacy is a core variable in your model, it would be useful to include more detailed discussion on this variable in the Introduction (e.g., be specific about how self-efficacy predicts adherence e.g., does lower or higher self-efficacy predict better adherence? How does lower/higher self-efficacy result in better adherence? What is its relationship with impulsivity?). It would also be helpful to specifically define and discuss diabetes-specific self-efficacy. Reference previous research and also justify why it has been proposed as a mediator between impulsivity and your outcome variables.

***More information about the relationship between self-efficacy and adherence is now provided, with references. In addition, we have included an example question from the self-efficacy for diabetes (SED) scale. Later in the Introduction we introduce the Hadj-Abo et al. (2020) study which provides additional justification for using self-efficacy as a mediator. In terms of how self-efficacy predicts adherence, this is still an open question. We don’t believe any model has adequately shown a psychological mechanism that explains this relationship, even as a mediator.***

Lines 44, Lines 60 etcetera – Inconsistent capitalization of Type 2.

***“Type 2” has been standardized throughout the manuscript.***

Provide a link between delay discounting and impulsivity so it is clear to the reader why you have introduced DD after mentioning “different impulsiveness constructs” e.g., is DD a behavioural measure of impulsivity? It may also be useful to specifically discuss how DD differs from the facets of impulsivity measured by the BIS-11 and why this distinction is useful.

***We have provided a brief discussion about how delay discounting and the BIS-11 typically show little association with each other, suggesting that they are measuring different aspects of impulsivity. We have inserted “one aspect of impulsivity” before delay discounting to predict the juxtaposition between delay discounting and the BIS-11.***

Provide a reference for this statement if available.

***This was a hypothetical statement – no reference is available.***

Define what HbA1c is for readers who may not be aware of this term.

***HbA1c has now been further defined.***

It would be helpful to provide more discussion as to why DD is associated with some diabetes management behaviours (e.g., non-adherence medication) but not others (e.g., diet adherence). Was this due to methodology, the study sample (e.g., adolescents with Type 1), or particulars of the DD task?

It would also be helpful to include studies that have investigated these relationships in participants with prediabetes (if available) and highlight any differences (or expected differences) in findings between these participant groups.

***There is very little systematic research in this area (i.e., directly comparing different methodologies/measures/participant samples). Thus, we chose not to speculate on this issue in the Introduction. The Discussion section is a more appropriate place for this level of analysis.***

***We have added a brief summary of a recent study (Epstein et al., 2020) that explores how glycemic control, medication adherence and delay discounting are related in individuals diagnosed with prediabetes.***

Reword this sentence to state the study examined the relationship between DD and non-adherence and poor glycemic control within a sample of adolescents with Type 1 diabetes (i.e., not between DD and diabetes).

***This sentence has been reworded.***

Update wording throughout manuscript to ensure person-first language (i.e., adolescents with Type 1 diabetes, not diabetic adolescents). See Dickinson et al. (2017) for guidance on this.

***The manuscript has been updated to use first-person language for individuals diagnosed with diabetes.***

I suggest changing the wording of this sentence to “….is frequently used as a self-report measure of impulsivity”

***The wording has been changed in this sentence.***

It would be helpful to mention in this paragraph that BIS-11 includes several subscales measuring different facets of impulsivity. Define these facets and discuss how they may/may not be related to diabetes self-care adherence. While BMI is a risk factor for diabetes, I believe this section could be much improved by including a summary of previous research specifically investigating the relationship between BIS-11 subscales (and other related measures of impulsivity if applicable) and diabetes related self-care adherence behaviours and outcomes. For example, Hadj-Abo et al., (2020) recently published a study investigating the mediating role of self-efficacy on the relationship between impulsivity and diabetes self-management. This study contains many of the same variables as the current manuscript so the findings from this research would be important to discuss in this Introduction.

***Unfortunately, this literature doesn’t exist, except for Hadj-Abo et al. (2020). Thus, a comprehensive discussion about differential associations between subscales and self-care adherence begins and ends with those results. We have now included a discussion of this article. Our study appears to be the second study to link these constructs together.***

Provide a reference for “a similar construct to impulsiveness” and be specific about its relationship with impulsivity. It should be clear why you are discussing this construct in the context of impulsivity, particularly as you have not measured it separately in the current study.

***This paragraph was deleted, as we did not explicitly measure ‘self-control’ in any way similar to these cited studies.***

Both studies mentioned in these lines have included an adolescent sample and discussed how the findings are relevant to self-care management for that particular population (i.e., parent observation). Given that research suggests adolescence is a developmental period characterised by increased impulsivity and risk-taking behaviours, it may not be appropriate to extrapolate the findings from these studies to the adult participants used in the current study.

***While adolescence differs in many ways from adulthood, the results for adolescents diagnosed with Type 1 diabetes were consistent with those for adults diagnosed with either Type 2 or prediabetes. Thus, we are comfortable keeping this information in the Introduction.***

Change “impulsiveness levels” to “impulsivity”.

***Done***

Materials & Methods

*Participants*

Provide an explanation as to why only participants over the age of 30 were recruited (particularly as many studies mentioned in the Introduction recruited adolescents)

***This explanation has been included in the Participants section.***

Demographic Descriptive Table – It would be helpful to split these demographics by participant groups and include a % column.

***Table 1 now has participants separated by diagnosis and also includes the percentage for each demographic variable.***

*Measures*

Include the rating scale, scoring information and brief description of psychometric properties (with references) for each measure.

***The rating scale, scoring information and a brief description of the psychometric properties for each measure have been added, with supporting references.***

Capitalize subscale titles e.g., the Control Problem subscale.

***Done***

Suggest rewording “the scale was normalized” to something that better represents what happened (e.g., “As the distribution of the Barrers to Testing Scale was positively skewed, the data was transformed using….”).

***This sentence has been reworded to be more descriptive.***

*Analyses*

Describe what software was used for analyses and reference accordingly (e.g., SPSS v.27; R; MPlus).

***The software that was used is more specifically described and referenced.***

Include mention that a Mann-Whitney U test was also performed on variables that were not normally distributed.

***Done***

Given the relationship between impulsivity and BMI/obesity (and the higher rates of BMI in populations with diabetes), it may be appropriate to consider including BMI as a covariate on the path models.

***The Path Models now include BMI as a covariate, whereas DD has been removed (see below).***

Results

Specify if there was any missing data and the procedures that were followed to account for it.

***All participants successfully completed all questions, therefore there was no missing data. This is specified in the Participants section.***

Include M & SD for BIS-11 total score and range of scores for all measures.

***The mean, standard deviation, and ranges have been included for all measures, including the overall BIS-11 scores.***

To assist the reader, report the results from the independent sample t-tests within this table (including the non-significant findings) and then comment on these findings in text. Effect sizes for these tests may also be useful for determining how meaningful the significant differences are.

***The independent sample t-tests have been included in the Table, along with Cohens d for those two differences that were statistically significant (Diet and Self-Efficacy).***

Clarify why DCP understanding was reported as N/A in the table.

***N/A has been clarified in Table 2 for the reader.***

Refer to table 3 in text before commenting on the results.

***Table 3 is now mentioned in the text before describing the results.***

Clarify if these relationships were found in both groups separately or if those were combined to get these results. If the groups were combined, please justify this action. Were the relationships the same if the groups were separated?

***As outlined in Table 3, Pearson zero-order correlations were performed for each group (Type 2 or prediabetes) separately. This has been clarified in the text.***

Report all correlations (including non-significant relationships such as BIS-11 & DD, and the relationships between self-efficacy and impulsivity measures) within this table.

***The Table has been updated to include correlations between the BIS subscales and SED.***

Report relationships between impulsivity measures and self-efficacy and comment on these in text.

***The relationship between all of the impulsivity measures (DD + BIS subscales) has been added to the Table and described in the text.***

Inconsistent use of scale names and constructs measured in text. Use names of specific subscales/measures in text to describe the relationships.

***The scale names and constructs have been more accurately presented in the Results section.***

Does this refer to the total BIS score? Report these correlations in the table.

***Table 3 presents each BIS subscale and the correlations with other self-care behaviors, self-efficacy and delay discounting. The rationale for using the overall BIS scores for the path analyses is included in the text.***

As stated earlier, include results of t tests in Table 3 and comment on these tests in text. Include statistics for non-sig. relationships.

***As Table 3 presents Pearson zero-order correlations, there are no t-tests to report. Significance (p < 0.05) is demarcated by asterisks.***

Path Models

Given the aim of this study was to examine the relationships between different impulsivity constructs, and that different correlation relationships between BIS-11 subscales and self-care/management problems were found, it is unclear why a total BIS-11 score (rather than subscales, specifically attention & non-planning) was used in the path model.

***Psychometric evaluations of the scale tend not to support its subscales and suggest a single factor structure provides a better fit to data (e.g., Steinberg et al., 2013). This justification is now included in the manuscript.***

In addition, as DD was not correlated with any model variables in this study it is unclear why this variable was included in the path models as a predictor of these outcomes. It may be more appropriate to consider using simple mediation models with the variables that have demonstrated significant relationships.

***We have removed DD as a variable in both Path Models, as it was not significantly correlated with any other theoretically important variables. Instead we have included BMI as a covariate in these new Path Models.***

I am also concerned that the sample size for each group may be too small to test a path model of this size. It is typically recommended to have a minimum 20 participants per parameter for SEM. While it may be a difficult population to recruit, it is important to highlight this limitation in the Discussion or use a different type of analysis.

***We have added sample size as a limitation in the Discussion section.***

It is inappropriate to call a non-significant relationship “marginally significant”. Remove this phrase from the manuscript (including from the Abstract and Discussion) and provide a correct interpretation of these relationships.

***“Marginally significant” has been removed throughout the text. These analyses are now classified as non-significant for interpretation.***

Discussion

There is a strong sense of repetition of the results section within this Discussion. While providing a summary of the results is important, please also include an interpretation of these findings in the context of previous research and the population sampled. Including a clinical implications section would be a valuable addition.

***We re-analyzed the relationship between impulsivity, self-efficacy, and self-care behaviors without DD, and with BMI as a covariate instead. We have included more interpretation of these findings. In addition, we have added a short section at the end of the Discussion section that touches on clinical applications for the current results.***

Include a discussion on how the findings from this study supported/did not support the hypotheses.

***We have included in the Discussion section descriptions of when our hypotheses were either supported, partially supported, or not supported.***

Please include a clear limitations and future directions section.

***A limitations section has been added to the end of the Discussion section.***

Remove text in brackets.

***Done***

Use third-person language. Check the rest of manuscript for this language as well.

***The manuscript has been edited to use third-person language.***

It is unclear why this paragraph has been included here. Is this paragraph being used to explain why there are often mixed findings when studies have used DD with populations with diabetes? Please provide some clarity and supporting references, and an explanation as to why other commodities (e.g., food) may be more appropriate.

***This paragraph has been updated to describe possible reasons why DD using money as the primary reward may not be associated with diabetes self-care behaviors.***

“an open question” is quite vague. Please clarify and expand the statement about causal status of self-efficacy. How does this study contribute to this status?

***This statement has been expanded to highlight the limitations of self-efficacy in this and other studies.***

These relationships were also not found in participants with prediabetes.

***This statement has been amended to reflect this finding for both groups.***

Type 2 diabetes is not a health behaviour.

***Type 2 diabetes is now described as a heath consequence.***

Provide a brief reason why DD results should be interpreted with caution as this was not immediately clear from the discussion.

***The limitations section now provides multiple reasons why the DD finding should be interpreted with caution.***

Delete “relatively robust”. This is term is quite vague and it is unclear what is meant by this statement.

***Done***

Rather than just suggesting replication, provide a more specific example e.g., longitudinal evaluation in larger sample etc?

***A more specific future direction was suggested in the text.***

Reviewer #2:

Specifically, in my opinion the introduction in too long. Please try to reduce it. For example, you could omit the general paragraphs on diabetes and prediabetes. In my opinion, these are well known concepts.

***Unfortunately, this is at odds with Reviewer #1 suggestion that we include more details about health complications, measurement, etc. We have tried to include this information, but without expanding on the well-known concepts too much.***

However, I'll briefly cite some paper examining the relationship between diabetes and psychopathology (especially the relevance on specific personality disorders in individuals with diabetes or ???diabetes). Some of these personality disorders are characterized by impulsiveness and may probably influence the brittleness of diabetes.

***The articles on brittleness of diabetes only focused on those patients diagnosed with Type 1 diabetes. Therefore, we are unsure whether this addition will aid in an understanding of relationships between impulsiveness and Type 2/prediabetes. This information may fit in the clinical implications section where we outline the role psychological constructs may play in remediating self-care adherence. However, this still feels somewhat ad hoc. For these reasons, we have chosen not to include brittleness in the current manuscript revision.***
