# Supplementary material for: Associations between impulsivity and self-care adherence in individuals diagnosed with Type 2 or prediabetes
Source: PLoS One. 2022 Mar 10;17(3):e0263961. doi: 10.1371/journal.pone.0263961 (PMC8912230; doi:10.1371/journal.pone.0263961)
Supplement: Attachment — Submitted filename: PONE-D-21-06140 R1 Response to Reviewers 12-19-21.docx [file pone.0263961.s003.docx]

PONE-D-21-06140R1

Associations between impulsiveness and self-care adherence in individuals diagnosed with type 2 or prediabetes

PLOS ONE

We would like to thank the Reviewers and Editor for their careful re-review of our manuscript. Per their comments, offer our response (in ***italicized bold*** font) to their suggestions in our revised manuscript:

Reviewer #1:

Abstract:

Lines 27-30: This is a very long sentence and suggests that both impulsivity measures have not been measured in the same study before (regardless of participant sample). This is not the case (when referring to participant samples beyond diabetes). I would suggest deleting “participants, or for” and specifying that delay discounting has not been measured in individuals with Type 2, while neither delay discounting & BIS have been measured in pre-diabetes (if that is the case).

***This sentence has been broken up to make it easier for the reader to understand that this is the first study to measure both impulsivity constructs in the same participants diagnosed with either Type 2 or prediabetes.***

To clarify your findings, It would be useful to also mention in the abstract that the choice-based personality measure was not associated with outcome variables so as to really support your final sentence in the abstract (that “how specific impulsivity constructs ….”)

***The lack of an association between delay discounting and self-care has been made more explicit.***

Lines 37-39: The final sentence of this abstract is somewhat misleading. Your study highlights what specific impulsivity constructs may be more useful to target in interventions but not how these constructs can be incorporated into interventions. I recommend rewording this sentence, so it more accurately reflects your study.

***This has been clarified.***

Introduction:

Line 56: Move the word “can” to after “requirements that”

Line 69: Insert commas as such: “impulsivity, such as a lack of inhibition, also”

Line 70: Include a comma after the last bracket

Line 112, include a comma after references

Line 95-96: I think it might be helpful to include a brief overall summary about what the consistent findings mean in relation to self-care, so it is very clear to the reader. Something like “Overall, the findings of these studies suggest that higher levels of impulsivity, as measured with the DD task, are associated with poorer diabetes self-care adherence. However, the findings…..”

Line 103: specific how BIS-11 was related to BMI e.g., positive, negative relationship (just so it is very clear to the reader).

Line 116: Due to a run on sentence, delete “like other important heath behaviours”. If you want to keep this in the introduction, I suggest including this comment as a separate sentence.

Line 117: insert comma after “studies”

Line 123: replace “for” with “in”

***All of the suggested edits in the Introduction have been made.***

Materials & Methods:

Line 196: Reword sentence to “Some items were reversed scored.”

Line 208: Are the authors referring to internal consistency and a valid measure of impulsivity here? Please report alpha values (or equivalent) are per previous measures in this manuscript, and specify what is meant by ‘valid’

***The consistency we referenced is response consistency (R^2^), whereas the type of validity we referred to is criterion validity. The magnitude effect is ubiquitous in delay discounting research. Thus, this is one criterion used to establish validity.***

Results:

Please ensure your Tables align with PLOS formatting requirements.

***All 3 Tables have been double-checked for formatting requirements.***

There is still inconsistent use of scale/subscale/measures in text/tables. E.g., see table 2, Lines 241-245, Lines 348-249.

***Scale/subscale/measures have been modified throughout the text to be more consistent.***

Lines 259-273 and Table 3. This section should come before correlations and path models as it provides further justification as to why you have chosen to analyze these groups separately. Table 3 should be introduced in text prior to appearing.

***Table 3 is now Table 2, and vice versa – there is now a introduction to Table 2 (formerly Table 3) before it appears in the text.***

Table 3: Reconsider the layout of the final column in this table. Not all rows in this table at t-tests, and the header of this column does not represent every value reported underneath it.

***Cohen’s d has been removed from the last column of the Table and an additional note about the Mann-Whitney test for DCP Control is made in the Results section. We chose to keep the Mann-Whitney statistical result in the same column as the t-tests as adding an additional column for one test seems like a waste of space.***

Table 2: Please include the total BIS-11 score in this correlation table and discuss in text.

***The total BIS-11 scores have been included into Table 3 (formerly Table 2). The significant positive correlation between DD and total BIS-11 scores for individuals diagnosed with prediabetes has been described in the Results section.***

Table 2 and Lines 227-256: Based on the authors’ justification in Lines 220-222, it is unclear why the BIS-11 subscale correlations are reported, and total score has been omitted here. The lack of relationship between some subscales of this measure for Type 2 (as reported in Line 231) and the differential relationships between these subscales and self-care adherence and self-efficacy appear at odds with this justification. As a reviewer, I am still not convinced that it is appropriate to use the total BIS-11 score in the path analyses. Theoretically, it makes sense as to why significant negative correlations are noted between BIS11 Non-Planning/Attention and these outcomes. Please ensure adequate and clear justification for this decision process is provided, beyond just the one study suggesting a single factor structure.

***We have an additional citation showing the psychometric validity for the Total BIS-11.***

Path Models:

Capitalize Type 2 in Line 279 and check the remaining manuscript for consistency.

Lines 283 & 287: For consistency, report “controlling for BMI” the same across these figure notes.

Lines 280 & 289: Delete ^p < .10 as this is not used in the figures.

***These changes have been made.***

Please provide justification for including BMI as a covariate as this appears to be a new addition since the first draft. From your results, it appears that BMI is not correlated with your outcome variables, so again, it is unclear why this covariate was included. Are the path models the same if you do not include BMI as a covariate? If so, report the path models without the covariate and include a note that the same models were run with BMI as a covariate, but findings did not differ (if justification for BMI covariate is included).

***Adding BMI as a covariate was suggested by Reviewer 1 in the last round of reviews. An additional justification (with citation) was added. We have re-calculated the path analyses with and without BMI as a covariate. When BMI is omitted as a covariate, there is only one change in the path analyses. This is for individuals diagnosed with prediabetes and is described in the text.***

Discussion:

To reorientate the readers, it is helpful to restate the hypotheses at the beginning of the discussion. Include a brief statement of the hypotheses following the first sentence in the discussion. This should be the first paragraph, then flow onto the findings.

***The hypotheses have been restated at the beginning of the Discussion section.***

Use third-person language (e.g., Lines 332, 336) and check the remaining manuscript for this.

Line 328: replace ‘predictive’ with ‘correlated’ to more accurately represent the data analysis and findings.

Lines 328-331: Discussion about DD might be better suited if it were included with the content in Lines 378-406

Line 335 – replace ‘this’ for ‘the’

***These changes/double-checks have been made.***

Lines 332-346: Overall, this paragraph would benefit from a re-write. The discussion bounces between the findings of Hadi-Abo and the current study’s results and hypotheses which makes it somewhat difficult to follow. I would suggest this type of structure: State the results. State how the results support/do not support the hypotheses. State how this relates to previous research. Explanation of findings in the context of the hypotheses and previous research. Be careful to avoid run-on sentences and ensure appropriate grammar (particularly the use of commas).

***We have reorganized this paragraph to increase readability.***

Line 344-346: This concluding sentence is too casual (i.e., replace “Perhaps there are” with “There may be”) and does not provide an explanation as to why self-efficacy did not mediate. It might be useful to hypothesise why this result occurred based on your knowledge of the literature, the constructs, and this study’s findings.

***We have added a hypothesis (and citation) to why we believe self-efficacy did not mediate the outcome.***

Lines 347-348: This sentence requires a slight re-write as it contains grammatical and word choice errors. The connection between this sentence and the current study’s results are unclear, please provide the link so it is clear to the readers. Something along the lines as “The current study aimed to address this gap in the literature…” would likely suffice.

***This change as been made.***

Lines 347-363: Similar to the feedback provided about Lines 332-346, this paragraph would also benefit from a re-write. At times, it was unclear if the authors were discussing the results related the pre-diabetes, or Type 2, or both (e.g., Lines 354-357)

***Like the previous paragraph, this paragraph was reorganized to increase readability.***

Line 360: Please clarify what the authors mean by “not high enough to impact…”. By scoring lower on self-efficacy, this would imply less adherence/self-care (at least based on Type 2 findings). It is unclear why scoring lower on this measure would result in a non-significant relationship. The significant difference between self-efficacy between participant groups implies that Type 2 have higher levels of self-efficacy compared to prediabetes – is this because they have likely spent more time engaging in these behaviors in the past? This would be important to explore in the discussion as it is likely an important target for clinical intervention (at least for Type 2)

***This sentence has been clarified and expanded upon.***

Line 367: As stated in the previous feedback, please remove the term “marginally significant” and provide the correct interpretation of results.

***This term has been removed.***

Line 337: Remove impulsivity. By the definition of personality, impulsivity is unlikely to be a treatment-modifiable factor. Given impulsivity is the same across these participant groups, longitudinal research should focus on factors that are likely to change over time and with treatment (such as self-efficacy and adherence).

***We have removed impulsivity. However, there is ample evidence that personality traits (such as impulsivity) can change over time within bounds. Additionally, DD has been decreased repeatedly using the episodic future thinking (EFT) paradigm.***

Lines 384-387: This is a run-on sentence. Consider rephrasing and writing in more succinct sentences.

Line 390: Delete “To date, no study has done this.”

***These last two changes have been made.***

Reviewer #2:

***We thank Reviewer #2 for clarifying why he thinks brittleness would be important for including in those individuals diagnosed with Type 2 diabetes. We will seriously consider including a measure for cluster B personality traits in a future study involving diabetes self-care and adherence.***
